# Homemade Glove Port for Single-Incision Pediatric Endosurgery (SIPES) Appendectomy—How We Do It

**DOI:** 10.1055/s-0038-1667140

**Published:** 2018-07-24

**Authors:** Illya Martynov, Martin Lacher

**Affiliations:** 1 Department of Pediatric Surgery, University of Leipzig, Leipzig, Germany

**Keywords:** children, glove port, appendicitis, single-incision pediatric endosurgery

## Abstract

**Introduction** Single-incision pediatric endosurgery (SIPES) for the treatment of acute appendicitis in children has recently gained popularity due to its advantages including minimization of postoperative scars or less incisional pain. The principal disadvantages of SIPES include the limited degrees of freedom of movement and high health care costs. To overcome these issues, some surgeons have reported to use noncommercial ports for SIPES appendectomy.

**Case Report** In this report, we present a case of a 10-year-old female patient with acute appendicitis undergoing SIPES appendectomy using own homemade glove port and straight rigid instruments.

**Conclusion** SIPES appendectomy using the glove port is a low-cost alternative to commercially available port systems. It is easy to set up and use.

## Introduction

Single port access (single-incision pediatric endosurgery [SIPES]) for laparoscopic appendectomy is increasingly being used. 1 2 3 However, special ports to perform SIPES appendectomy may not be affordable in developing countries. 4 5 6 Moreover, commercially available SIPES ports are known to have a limited range of motion of the surgical instruments. 7 8 Therefore, alternatives to overcome both obstacles are warranted. In this technical report, we present our way to set up and carry out SIPES via a homemade glove port.

## Case Report

**Fig. 1:**
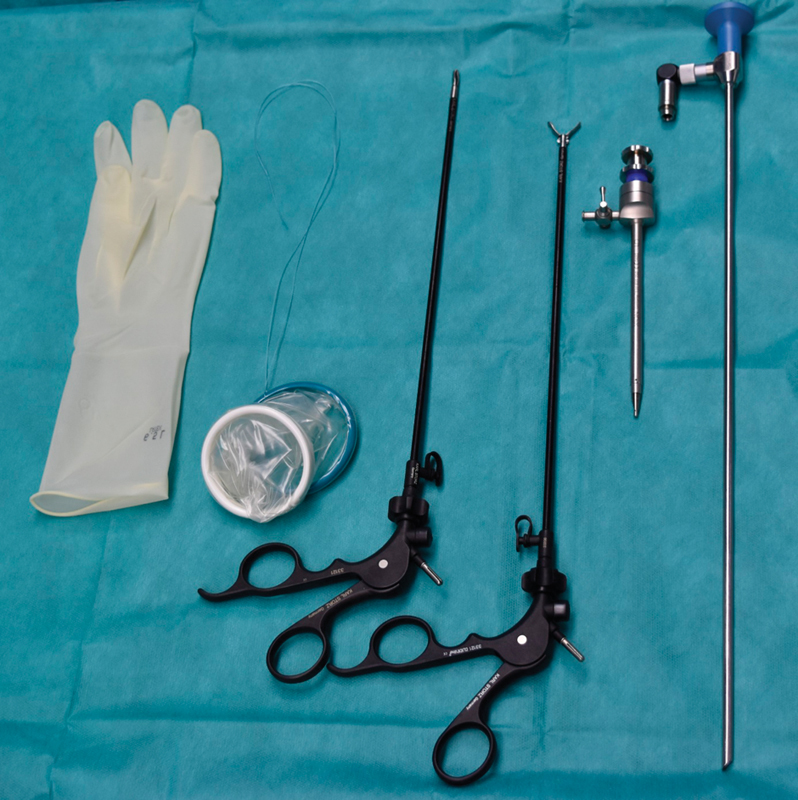
Equipment used for single-incision pediatric endosurgery with glove port.

**Fig. 2:**
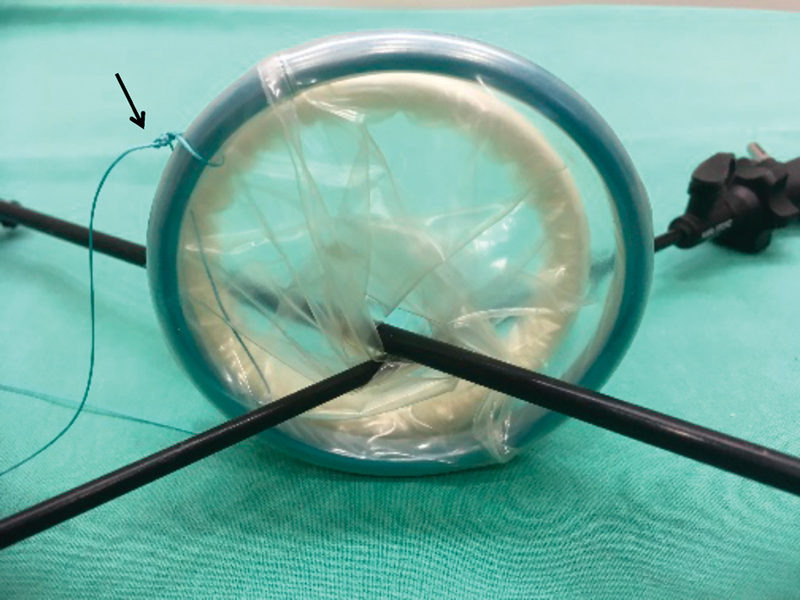
Hitch stich to facilitate removal of the Alexis-Port.

**Fig. 3:**
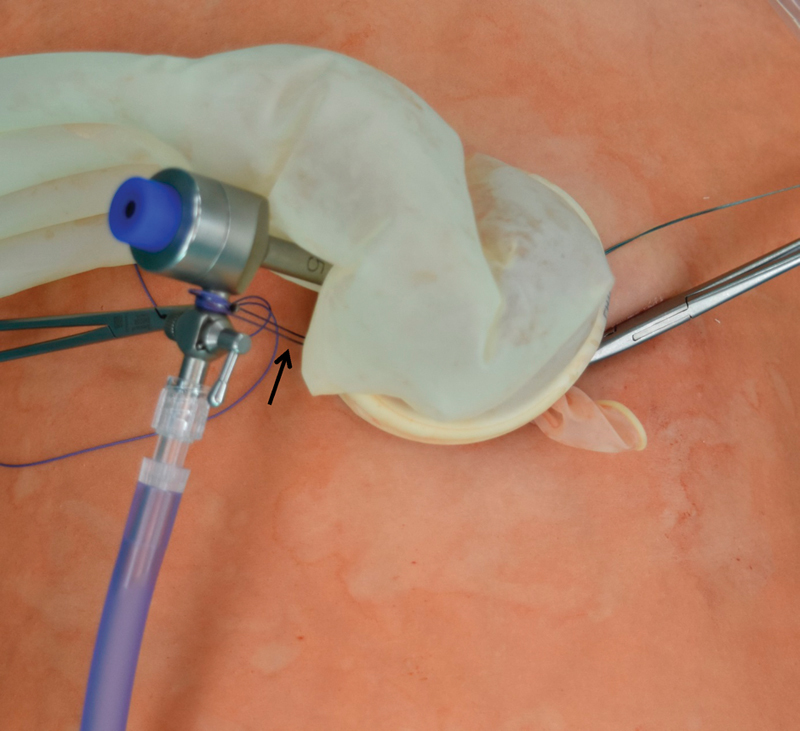
The fixation of 5-mm trocar between the trocar valve and the Alexis-Port.

A 10-year-old female presented to the emergency room with diffuse abdominal pain for 1 day, and two episodes of vomiting. The clinical and laboratory findings were consistent with appendicitis. Therefore, the girl was taken to the operating room and surgical table set up for SIPES appendectomy, while glove port was prepared ( Fig. 1 ). A 2-cm vertical incision was made in the fascia underlying the umbilicus to enter the peritoneal cavity. A wound retractor (Alexis, Size XS, Applied Medical Resources Corp., Rancho Santa Margarita, CA) was placed directly through the fascia, and a 6.5 size latex sterile powder-free surgical glove was connected to it ( Fig. 2 ). The thumb of the glove was cut off and a 5-mm trocar (Karl Storz, Germany) was introduced in the abdomen for CO _2_ insufflation and introduction of the monopolar hook and tied to the wound retractor to prevent dislocation ( Fig. 3 ). A 5-mm 45-cm scope (Stryker Endoscopy, San Jose, CA) was connected to the light cord using a 90° angulated light adapter (Karl Storz) and introduced through a 2-mm incision in one of the finger tips. With standard reusable 5-mm straight laparoscopic instruments, introduced in the same technique as the camera, the appendix was identified, and the mesoappendix divided. The appendix was grabbed, the capnoperitoneum was deflated, and the appendix exteriorized and amputated over a polyglactin suture ligation extracorporeally. The fascial incision was approximated with a running 2–0 polyglactin suture. Finally, the skin incision was closed using interrupted subcuticular 4–0 poliglecaprone sutures ( Video 1 ). Histological examination confirmed the diagnosis of appendicitis. There were no intra- or postoperative complications. The patient was discharged on postoperative day 2.

**Video 1**

Creation of highly effective glove port and subsequently appendectomy procedure using the single-incision pediatric endosurgery (SIPES) technique. Online content including video sequences viewable at: www.thieme-connect.com/products/ejournals/html/10-1055-s-0038-1667140-EJPSR-18-0378-v1.mp4 .

## Discussion

**Fig. 4:**
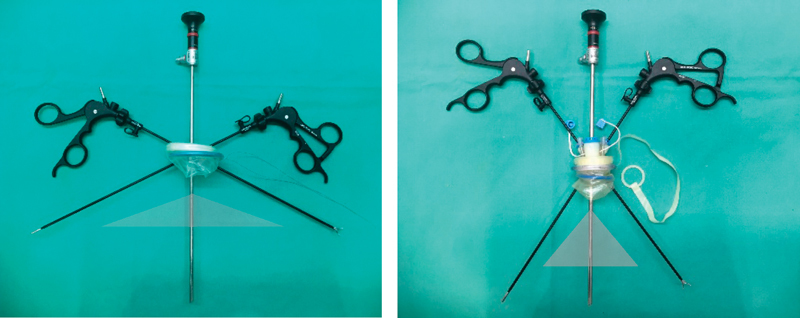
Comparison of ranges of motion between homemade glove port and a commercially available device (Tri-Port, Olympus, Center Valley, PA).

SIPES provides additional advantages over traditional 3-port laparoscopy such as improved cosmetic and the ability to perform multiple procedures without the need for additional ports. 2 9 10 Glove ports rather than traditional ports can be used as a cost-effective method with improved ergonomics to circumvent the limitations of the previous single ports. 4 11 12 13 14 Furthermore, the elasticity and pliability of the surgical glove port compared with commercially available ports allows reasonable range of movements and achieves enough triangulation ( Fig. 4 ). This has been also shown by others. 14 However, despite several advantages of homemade glove port, the application for SIPES is not approved and can be an issue for some medical institutions. For patients with a potential for developing a latex allergy, nonlatex surgical gloves should be used. The current expenses for SIPES ports range from 318 to 1,378 USD 15 16 17 compared with glove ports based on equipment available for around 30 euro. 4

With the aim to further improve the technique of setting up the glove port, we established the following modifications:

1. The fixation of a 5-mm trocar to the wound retractor prevents dislocation and allows the introduction of sharp instruments through the port as the tip of the port cannot violate the glove.
2. All other instruments (2nd grasper and the camera) can be introduced through the 2-mm incisions in the finger tips, therefore time-consuming connection of special cannulas or trocars as suggested by others is not necessary. 14 18
3. The hitch stich, the wound retractor, allows easy removal at the end of the procedure.
4. The bariatric scope in combination with the 90° angled light connector (Karl Storz) avoids clashing of instruments and the instruments and the telescope/camera head.

## Conclusion

SIPES appendectomy using a homemade glove port is a low-cost alternative to commercially available ports and it is easy to set up and use. Moreover, the high flexibility of the glove and the wide range of motion of the instruments make SIPES more comfortable than using most SIPES ports currently available in the market. Therefore, this device is a good option even in highly equipped laparoscopic centers.

## Notes

New Insights and the Importance for a Pediatric Surgeon The surgical equipment to perform single-incision pediatric endosurgery (SIPES) does not have to be expensive. The glove port presented in this technical report is not only highly cost effective but allows a wide range of motion of the surgical instruments. Therefore, homemade glove ports should have a role even in highly developed countries.
